# Supplementary material for: Predictors for Uptake of Vaccines Offered during the Second Year of Life: Second Dose of Measles-Containing Vaccine and Meningococcal Serogroup A-Containing Vaccine, Ghana, 2020
Source: Vaccines (Basel). 2023 Sep 23;11(10):1515. doi: 10.3390/vaccines11101515 (PMC10611024; doi:10.3390/vaccines11101515)
Supplement: Supplementary file 1 [file vaccines-11-01515-s001.zip › vaccines-2551085-supplementary.pdf]

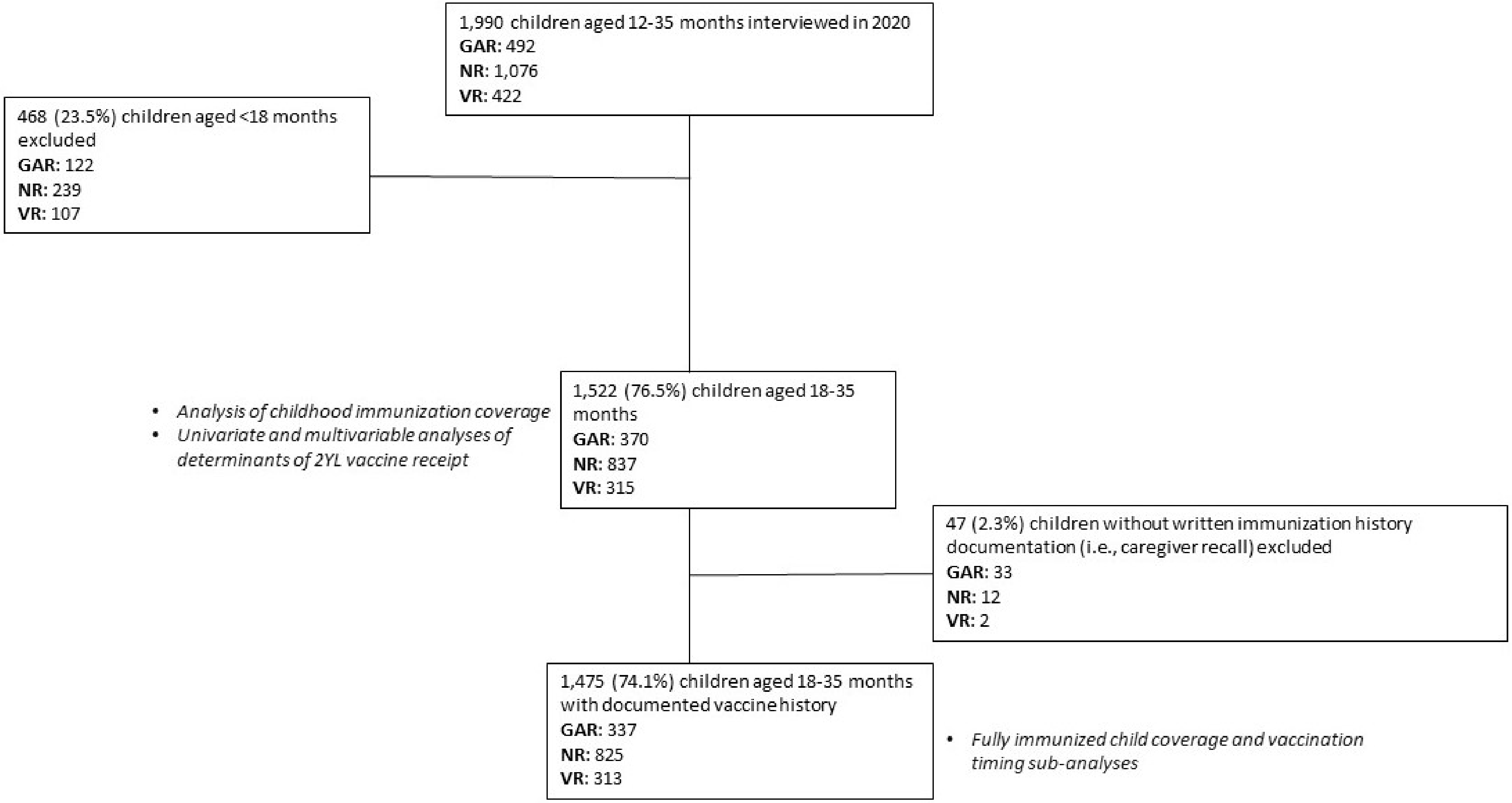

Supplementary Figure S1: Flowchart for selection of analytic samples.

Supplementary Table S1. Variables assessed as predictors of 2YL vaccine receipt

**Child age group (months)**
18-23
24-35
**Child's sex**
Female
Male
**Birth order**
First
Second
Third or more
**Maternal age (years)**
<25
25-34
≥35
**Paternal age (years)**
<25
25-34
≥35
**Maternal education**
Never attended school
Primary
Secondary or higher
**Paternal education**
Never attended school
Primary
Secondary or higher
**Maternal religion**
Other
Christian
**Maternal marital status**
Single/Divorced/Separated/Widowed
Married/Co-habitating
**Settlement type**

Urban
Rural
**Household size**
More than 6 members
6 or less members
**Caregiver has access to phone for text messaging**
No
Yes
**Caregiver received phone reminder for 18 mo visit**
No reminder received
Reminder received
**Child attends school/daycare**
No
Yes
**Child attends school/daycare offering routine immunization**
No
Yes
**Child's HBR reviewed at school**
No
Yes
**Fully immunized child by 12 mo**
No
Yes
**Caregiver knows someone with measles**
No
Yes
**Caregiver knows 2 doses are needed for full protection**
No
Yes
**Caregiver knows MCV dose should be given during 2YL**
No
Yes
**Caregiver knows MACV dose should be given during 2YL**
No
Yes
**Caregiver thinks more than one MCV dose is needed for full protection**

No
Yes
**Caregiver knows MCV doses are given at 9 & 18 mo**
No
Yes
**Caregiver knows MACV dose is given at 18 mo**
No
Yes
**Caregiver aware of need for 18 mo visit before 2nd birthday**
No
Yes
**Caregiver believes healthcare provider provides enough information on 2YL vaccine**
No
Yes
**Healthcare provider is caregiver's most trusted source of immunization information**
No
Yes
**Caregiver believes vaccines protect against VPDs**
No
Yes
**Caregiver believes vaccines are safe**
No
Yes
**Caregiver believes completely unvaccinated children are vulnerable to disease**
No
Yes
**Caregiver believes unvaccinated children can infect others**
No
Yes
**Caregiver believes 2YL vaccine is important for child**
No
Yes
**Caregiver believes 2YL vaccine is important for other children**
No
Yes
**Both father and mother involved in decision to vaccinate child**

No			
Yes			
**Caregiver believes 2YL vaccination is supported by spouse**			
No			
Yes			

Supplementary Table S2. Model output for the predictors of MCV2 receipt in Greater Accra Region with interaction term for school availability of RI services and settlement type**.**

	aOR	95 % CI	P value
**Child's sex**			
Female	REF		
Male	1.0	0.5–2.1	0.923
**Birth order**			
First	REF		
Second	0.7	0.2– 2.7	0.698
Third or more	0.3	0.2–0.8	0.016
**Maternal education**			
Never attended school	REF		
Primary	1.5	0.5–5.0	0.468
Secondary or higher	2.0	0.9–4.7	0.069
**Maternal age (years)**			
<25	REF		
25-34	2.4	0.5–11.4	0.265
≥35	2.8	0.8–9.2	0.087
**Maternal religion**			
Other	REF		
Christian	1.1	0.5–2.5	0.853
**Settlement type**			
Urban	REF		
Rural	4.7	0.8–28.9	0.094
**Attends school/daycare offering routine immunization**			
No	REF		
Yes	0.8	0.3–1.9	0.567

**Interaction term**	0.1	0.02–0.8	0.031
**Fully immunized child by 12 mos.**			
No	REF		
Yes	4.0	1.7–9.4	0.002
**Caregiver knows MR dose should be given during 2YL**			
No	REF		
Yes	2.4	1.2–4.9	0.017

Supplementary Table S3. Joint effects of settlement type and school RI service availability on MCV2 receipt among children aged 18–35 months in Greater Accra Region

	aOR	95% CI	P value
Urban school; no RI service available	REF		
Urban school; RI service available	0.8	0.3–1.9	0.567
Rural school; no RI service available	4.7	0.8–28.9	0.094
Rural school; RI service available	0.4	0.2–1.2	0.102
Joint effects were calculated from the model with the interaction term using the lincom post-estimation Stata command			

Supplementary Table S4: MCV2 coverage among children aged 18–35 months by availability of RI services at school and settlement type in Greater Accra region, Ghana, 2020

MCV2 dose received	Urban				Rural			
	Attends school/daycare offering routine immunization (RI)							
	No	Yes	Total	P value	No	Yes	Total	P value
No	83	22	105		12	3	15	
	37.4 (31.1,44.1)	23.1 (15.8,32.5)	33.2 (27.9,39.1)	0.011	24.6 (12.6, 42.5)	35.3 (23.9,48.6)	27.13 (16.5,41.3)	0.094
Yes	147	72	219		27	4	31	
	62.6 (55.9,68.9)	76.9 (67.5,84.3)	66.8 (60.9,72.1)		75.4 (57.5,87.4)	64.7 (51.4,76.1)	72.87 (58.7,83.5)	

Total	230	94	324	39	7	46
	100.00	100.00	100.00	100.00	100.00	100.00

First row shows the *unweighted counts* and second row has *MCV2 coverage with 95% CIs*
P value represents significance of design-based chi square test
